# Different Associations of Trunk and Lower-Body Fat Mass Distribution with Cardiometabolic Risk Factors between Healthy Middle-Aged Men and Women

**DOI:** 10.1155/2018/1289485

**Published:** 2018-02-04

**Authors:** Bin Wu, Jingshan Huang, Keisuke Fukuo, Kazuhisa Suzuki, Gen Yoshino, Tsutomu Kazumi

**Affiliations:** ^1^Open Research Center for Studying of Lifestyle-Related Diseases, Mukogawa Women's University, 6-46 Ikebiraki-cho, Nishinomiya, Hyogo 663-8558, Japan; ^2^Department of Endocrinology, First Affiliated Hospital of Kunming Medical University, Kunming, Yunnan 650032, China; ^3^School of Computing, University of South Alabama, Mobile, AL 36688-0002, USA; ^4^Department of Food Sciences and Nutrition, School of Human Environmental Science, Mukogawa Women's University, 6-46 Ikebiraki-cho, Nishinomiya, Hyogo 663-8558, Japan; ^5^Research Institute for Nutrition Sciences, Mukogawa Women's University, 6-46 Ikebiraki-cho, Nishinomiya, Hyogo 663-8558, Japan; ^6^Department of Diabetes and Endocrinology, Toho University Omori Medical Center, Omori-Ku, Omori-nishi 6-11-1, Tokyo 143-8541, Japan

## Abstract

The aim of this study was to assess whether the gender-specific pattern of fat mass (FM) distribution is related to gender differences in cardiometabolic risk factors. 207 healthy middle-aged Japanese were included in the study. We measured FM in the total body, trunk, and lower-body with dual-energy X-ray absorptiometry (DXA). The percentage of trunk FM (TFM) and lower-body FM (LFM) is noted as %TFM and %LFM, respectively. Other measurements included glucose and insulin during oral glucose tolerance test (OGTT), leptin, adiponectin, plasminogen activator inhibitor-1 (PAI-1), tumor necrosis factor-*α* (TNF-*α*), C-reactive protein (CRP), and systemic oxidative stress marker. Arterial properties were indicated by cardio-ankle vascular index (CAVI) and intima-media thickness (IMT) of the common carotid artery. The results showed that %TFM is higher whereas %LFM is lower in men than in women and men have a more atherogenic cardiometabolic profile. In both genders, %TFM (%LFM) is related to an unfavorable (favorable) cardiometabolic profile. In particular, the relation between %LFM and OGTT-derived insulin sensitivity index is stronger in women than in men. These findings suggested that in relatively healthy adults, android and gynoid pattern of FM distribution contributes to gender differences in cardiometabolic risk factors.

## 1. Introduction

Obesity is a widely accepted risk factor strongly associated with type 2 diabetes and cardiovascular diseases (CVD) [1]. Epidemiological studies have suggested that indirect measurements of body fat mass (FM), such as body mass index (BMI) and waist circumference (WC), are positively related to diabetes and CVD [1, 2]. This association might be explained by a high BMI and WC representing a large amount of body fat accumulation, especially in the abdominal region. However, indirect anthropometric indices do not count for the variation in body fat distribution, which can considerably differ for the same BMI across different genders, ages, and races [3–5]. Recently, computed tomography (CT) has been developed as a standard method to precisely evaluate abdominal fat distribution [6], but its high cost and exposure to radiation have limited the usage of CT in the investigation of body FM topography [7]. Furthermore, it is not feasible to measure the whole body composition by using CT scan which may cause massive radiation. On the other hand, dual-energy X-ray absorptiometry (DXA) provides a noninvasive, rapid, and accurate method for separating body mass into bone mass, fat mass, and fat-free mass, assessing both total and regional body fat contents with relatively low cost and low radiation exposure [8, 9]. It is thus extensively used as a tool for investigating the contribution of whole body FM distribution to CVD risk factors.

Body FM distribution in relation to lipid and glucose metabolism has been investigated in prior studies [8–16]. DXA-derived trunk fat mass (TFM or android FM) is strongly associated with intra-abdominal adiposity measured by CT or magnetic resonance imaging (MRI) [8, 9]. TFM is also strongly inversely related to insulin sensitivity measured by the glucose infusion rate during euglycemic-hyperinsulinemic clamp [10]. In contrast to TFM, lower-body fat mass (LFM or gynoid FM) was reported to have advantageous effects on glucose [12] and lipid metabolism [10, 14, 15] after TFM has been taken into consideration. Moreover, LFM adjusted for total FM was also reported to be related to a low risk of diabetes in Africans [13] and a low CVD risk in Swedes [16]. The potential role of adipokines and insulin resistance in mediating the protective effects of LFM has been discussed.

Whereas male gender is an established risk factor for CVD, it is not yet fully understood whether the different levels of cardiometabolic risk factors between women and men are related to the gender-specific pattern of FM distribution. In addition, it remains unclear whether opposite effects of TFM and LFM on metabolism can be further extended to an association between DXA-derived fat indices and direct atherosclerotic indices such as arterial stiffness and thickness. Therefore, the main goal of this study is to investigate DXA-derived body FM distribution and its relationship with various cardiometabolic risk factors including glucose and lipid metabolism parameters, adipokines, inflammation markers, systemic oxidative stress, and arterial stiffness and thickness in a middle-aged Japanese cohort. In particular, gender-specific effects of TFM and LFM on the cardiometabolic profile will be compared in detail.

## 2. Study Subjects and Methods

### 2.1. Subjects

A total of 148 female and 59 male adults were recruited as subjects. This study was approved by the Ethics Committees of Mukogawa Women's University, and written informed consents were obtained from all participants. The study was conducted in compliance with the Declaration of Helsinki (revised in October 2008) and the “Ethical Guidelines for Clinical Studies” issued by the Ministry of Health, Labour and Welfare of Japan (amended in December 28, 2004).

- All participants were Japanese residing in Hyogo prefecture.
- Subjects were excluded from the study if they were clinically diagnosed in various diseases: acute or chronic inflammatory diseases and endocrine, cardiovascular, hepatic, and renal diseases.
- Subjects who had hormonal contraception were excluded.
- Regular cigarette smokers and/or alcohol drinkers were also excluded.
- No subjects were classified with metabolic syndrome according to the NCEP ATP III [17], IDF [18], or Japanese [19] criteria.

### 2.2. Anthropometry, Body Composition, and Fat Mass Distribution

Body weight, height, and WC were measured following the standard procedures, and then, BMI was calculated. DXA with a scanner (Hologic QDR-2000, software version 7.20D, Waltham, MA) was utilized to measure body mass distribution. The Hologic method used a three-compartment model of body mass and provided an estimation of regional FM, lean mass, and bone mineral. A scanned image of the whole body was divided into six subdivisions: head, trunk, and left/right limbs. The dividing borders between two subregions were differentiated by a line underneath the chin, a line between the humerus head and the glenoid fossa, and a line at the femoral neck, respectively. The trunk region included the chest and abdomen but excluded the pelvis. The lower-body region included the entire hip, thigh, and leg.

The percentage of TFM is noted as %TFM and calculated as (TFM/total FM) × 100%, and the percentage of LFM is noted as %LFM and calculated as (LFM/total FM) × 100%. Android and gynoid fat distributions were expressed as %TFM and %LFM, respectively.

### 2.3. Insulin, Glucose, and Insulin Resistance

Blood samples were obtained in the morning after a 12-hour overnight fasting. Oral glucose tolerance test (OGTT) was performed with 75 g glucose administration. Blood samples were taken at minutes 0, 30, 60, and 120 for glucose and insulin analyses. Plasma glucose was determined by the hexokinase/glucose-6-phosphate dehydrogenase method, with an interassay coefficient of variation (CV) that was less than 2%.

Serum insulin was measured by an ELISA method with a narrow specificity excluding des-31, des-32, and intact proinsulin, with an interassay CV that was less than 6%. Insulin resistance was determined by both (A) homeostasis model assessment (HOMA-IR) using fasting plasma glucose and insulin levels [20] and (B) Matsuda index (i.e., insulin sensitivity index (ISI)) using glucose and insulin levels during OGTT [21].

### 2.4. Lipids, Lipoprotein, and Apo Measurements

Serum lipids, including triglycerides (TG), total cholesterol (TC), and high-density lipoprotein cholesterol, were measured using an autoanalyzer (AU5232, Olympus, Tokyo, Japan). Apolipoprotein A-1 (ApoA1) and apolipoprotein B-100 (ApoB) were measured by commercially available kits using an Olympus autoanalyzer (AU600, Mitsubishi Chemicals, Tokyo, Japan). Low-density lipoprotein cholesterol (LDL-C) was determined using the Friedewald formula [22]. Small, dense LDL-C (sdLDL-C) was measured by a precipitation method described in [23]. Free fatty acid (FFA) was measured using enzymatic colorimetric methods (Wako, Neuss, Germany). Remnant-like particle cholesterol (RLP-C) was measured by an immunoaffinity separation method (RLP-C assay, Otsuka, Japan). Preheparin serum lipoprotein lipase (LPL) mass was measured by a sandwich ELISA using a specific monoclonal antibody against bovine milk LPL, as described by Kobayashi et al. [24]. A commercial kit from Daiichi Pure Chemicals (Tokyo, Japan, with an interassay CV = 2.8%) was utilized.

### 2.5. Adipokines, Inflammation, and Oxidative Stress Markers

Adiponectin was assayed by a sandwich enzyme-linked immunosorbent assay (Otsuka Pharmaceutical Co. Ltd., Tokushima, Japan). Intra- and interassay CV were 3.3% and 7.5%, respectively. Leptin was assessed by a radioimmunoassay (RIA) kit from LINCO Research (St. Charles, MO, with an interassay CV = 4.9%). Highly sensitive C-reactive protein (hs-CRP) was measured by an immunoturbidimetric assay with the use of reagents and calibrators from Dade Behring Marburg GmbH (Marburg, Germany, with an interassay CV less than 5%). TNF-*α* was measured by immunoassays (R&D Systems Inc., Minneapolis, MN, with an interassay CV less than 6%). PAI-1 was measured by an ELISA method (Mitsubishi Chemicals, Tokyo, Japan, with an interassay CV less than 8%). For statistical analysis, serum concentrations of hs-CRP and TNF-*α* below the limit of detection were assigned a value of 0.05 mg/L and 0.50 pg/mL (the lowest limit of detection), respectively. Systemic oxidative stress was evaluated by urinary creatinine-indexed 8-epi-prostaglandin F_2*α*_ (8-epi-PGF_2*α*_), a validated biomarker of systemic oxidative stress [25]. Urinary 8-epi-PGF_2*α*_ was measured in the first-voided morning urine sample with an enzyme-like immunosorbent assay (8-isoprostane EIA kit, Cayman, Ann Arbor, MI). Intra- and interassay CV were 7.5% and 9.2%, respectively. Urinary 8-epi-PGF_2*α*_ was indexed to creatinine as picograms per millimole creatinine.

### 2.6. Arterial Properties

Systolic blood pressure (SBP) and diastolic blood pressure (DBP) were measured with standard methods. Arterial stiffness was indicated by cardio-ankle vascular index (CAVI) (VaSera, VS-1000, Fukuda Denshi, Tokyo, Japan). CAVI is a recently developed index that reflects stiffness of the aorta and femoral and tibial artery [26]. CAVI involves the measurement of pulse wave velocity (PWV), but the effects of blood pressure are minimized. It has thus been validated to be a reliable screening tool for atherosclerosis [26]. Arterial thickness was evaluated by intima-media thickness (IMT) of the common carotid artery using an ultrasonic device (SDU-1100, Shimadzu, Tokyo, Japan). The maximum IMT was assessed at the far wall as the distance between the interface of the lumen and intima and the interface of the media and adventitia. The maximum IMT of two measurements done at each of the four-segment vessels was recorded on both sides and averaged for the left and right sides. The average (Ave IMT) and maximum (Max IMT) of the four IMT values were used for further analysis. Note that IMT was clinically used as an indicator of generalized atherosclerosis [27].

### 2.7. Statistical Analysis

Data were presented as mean ± standard deviation (SD). Due to deviation from normal distribution, C-reactive protein (CRP) was logarithmic transformed for analysis. Mean differences between females and males were compared by the nonparametric Mann–Whitney *U* test. Analysis of covariates (ANCOVA) was used to test the effects of body FM distribution on gender differences in cardiometabolic risk factors, where cardiometabolic risk factors, gender, and %TFM and %LFM were entered into a general lineal model as dependent variables, fixed factor, and covariates, respectively.

Multivariate linear regression was used to assess the significance of cross-sectional relations among %TFM, %LFM, and cardiometabolic risk factors both before and after adjusting for covariates. Variables were transformed to standard *Z*-scores (zero mean and unit variance). Regression results were reported as standardized *β* to facilitate a direct comparison. A standardized *β* of 0.1 indicated that every increase in the 1-SD independent variable may lead to a 0.1-SD increase in the dependent variable. Age, BMI, and WC were entered into the models as covariates, and all models were gender-specific to account for the sex interactions observed. The stability of the regression model was considered to be disturbed by multicollinearity if the variation inflation factor (VIF) was >10. VIF is a statistic used to detect how much the independent variables are linearly related to one another. VIF is calculated as 1/(1 − *R*^2^) for an independent variable when it is predicted by other independent variables that have already been employed in the mode.

Bivariate correlations of regional FM distribution and cardiometabolic parameters were evaluated by both Pearson's and Spearman's correlation analyses. These two methods gave practically identical results; therefore, only Pearson's correlation coefficients were presented. To compare the strength of correlation coefficients between women and men, *Z*-statistic was used. Pearson's correlation coefficients (*r*) were recoded to Fisher *Z*-transform (*Z_f_*). *Z*_*f*_ = 1/2ln((1 + *r*)/(1 − *r*)), and the differences in $Z\;value=\left(Z_{f1}-Z_{f2}\right)/\sqrt{\left(1/\left(N1-3\right)\right)+\left(1/\left(N2-3\right)\right)}$. *N* represents the sample size. *Z* values are approximately standardly normally distributed and used to determine the level of significance. A two-tailed value of *P* < 0.05 was considered significant. Statistics were performed with SPSS system 17.0 (SPSS Inc., Chicago, IL).

## 3. Results

### 3.1. Experimental Results

FM distribution and cardiometabolic characteristics of a female and male cohort are presented in Table 1. Note that

- “%total FM” in the table refers to the percentage of total FM in the total body weight (i.e., %total FM = (total FM/total body weight) × 100%);
- Mean ages of women and men are 49.8 years (ranging from 39 to 60 years) and 51.8 yeas (ranging from 38 to 64 yeas), respectively, and women and men had different FM distributions (*P* < 0.001).

Compared with women, men had less %total FM but higher %TFM and lower %LFM. Gender differences in HDL-C, sdLDL, preheparin serum LPL mass, leptin, adiponectin, CRP, DBP, and CAVI were significant both before and after adjusting for %TFM and %LFM. To be more specific, gender differences were relatively insignificant before adjusting for ISI, but after FM distribution parameters were taken into account, mean difference of Matsuda index became much more significant (*P* < 0.001). On the contrary, gender difference in TG lost its significance after adjusting for covariates. Gender differences in RLP-C, PAI-1, and SBP were significant and consistent after adjusting for %LFM but disappeared after adjusting for %TFM.

Results of multiple linear regression analysis of %TFM, %LFM, and cardiometabolic risk factors are shown in Table 2. In both genders, %TFM (%LFM) was negatively (positively) associated with Matsuda index and adiponectin after age, BMI, and WC were adjusted. The controversial pattern of association was observed for HOMA-IR, TG, and ApoB in women and for TG and RLP-C in men. In women, %TFM and %LFM were significantly but oppositely related to HDL-C, sdLDL, ApoA1, leptin, PAI-1, CRP, TNF-*α*, and SBP, but the significances were weakened or even completely disappeared after multivariable adjustment. In men, the same pattern of associations was observed for FFA. %TFM was positively and consistently related to PAI-1 and TNF-*α* after adjusting for covariates in men, but not in women. This pattern of associations with PAI-1 and TNF-*α* was not found for %LFM in both genders. No significant relationships of %TFM and %LFM with urinary 8-epi-PGF_2*α*_, CAVI, and IMT were found in both genders.

**Figure 1:**
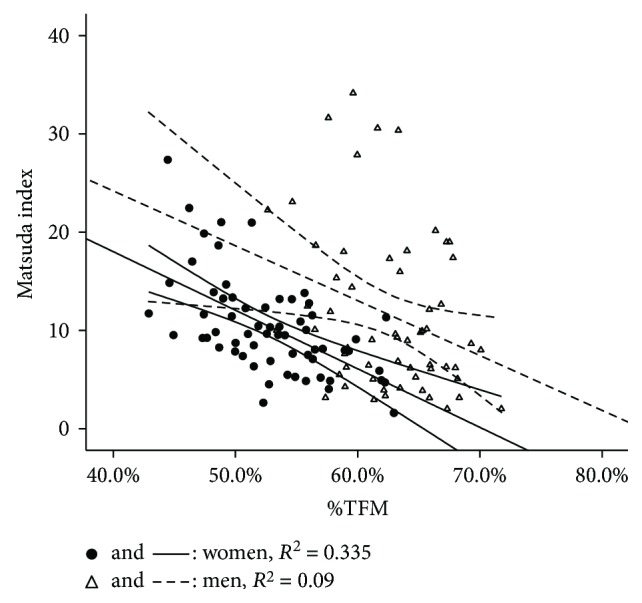
Regression lines of %TFM versus Matsuda index with 95% confidence interval in women and men.

**Figure 2:**
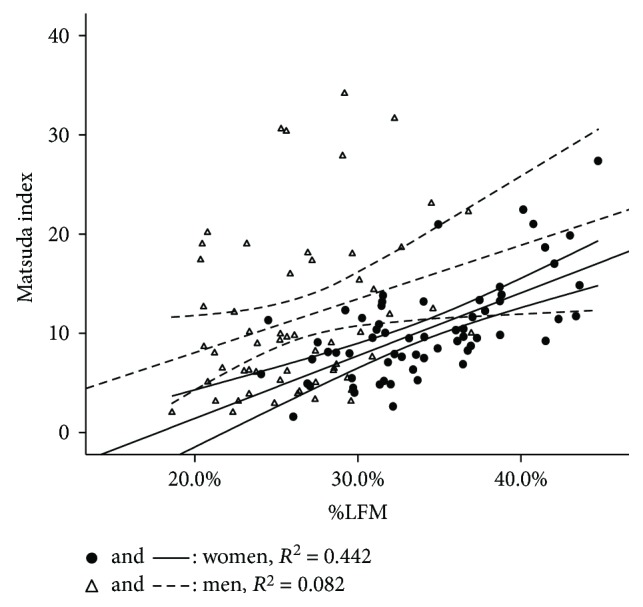
Regression lines of %LFM versus Matsuda index with 95% confidence interval in women and men.

Bivariate correlation analysis among %TFM, %LFM, and primary outcome variables (Matsuda index, triglycerides, and adiponectin) in regression analysis, along with the comparison of correlation coefficients between women and men, is presented in Table 3. Coefficients among %TFM, %LFM, and Matsuda index were stronger in women than in men (−0.579 versus −0.299 for %TFM, with *P* = 0.025; 0.644 versus 0.286 for %LFM, with *P* = 0.003). Compared with men, stronger association between %LFM and Matsuda index was observed in women after adjusting for age/BMI/WC (0.551 versus 0.214, with *P* = 0.011). There were no significant gender differences in correlations of %TFM and %LFM with triglycerides and adiponectin. Sex-stratified bivariate correlations among %TFM, %LFM, and Matsuda index (delineated by regression lines with 95% confidence interval) were illustrated in Figures 1 and 2.

## 4. Discussion

Identifying cardiometabolic risk factors in a healthy population before the onset of CVD is important for establishing primary preventive strategies. We aim to define the role of FM distribution in gender differences of various cardiometabolic risk factors in an adult cohort with relatively lower CVD risks. Our experimental results demonstrated that android fat distribution and gynoid fat distribution have significant but opposite correlations with insulin sensitivity, triglyceride, and adiponectin in both genders independent of age/BMI/WC. %LFM was related to a more favorable cardiometabolic profile in both genders. In particular, relation between %LFM and ISI was stronger in women than in men. On the contrary, %TFM was related to an unfavorable cardiometabolic profile in both genders. Additionally, %TFM was related to PAI-1 and RLP-C independent of BMI and WC in men but not in women. No associations were found between FM distribution and arterial property indices in both genders.

Well-known differences in FM distribution between genders were observed in this study. Despite their lower total fat percentage, men had 8% to 11% higher %TFM than women, whereas women had 6% to 9% higher %LFM than men. Due to the fact that more than 90% of LFM is located in the subcutaneous region [28], our findings suggested that women generally tend to accumulate fat mass in the subcutaneous compartment at a higher rate than men. Distinct gender differences in cardiometabolic risk factors were observed. Compared with women, male subjects exhibited an unfavorable cardiometabolic profile characterized by an atherogenic lipoprotein phenotype accompanying with hyperleptinemia, hypoadiponectinemia, higher blood pressure, and stiffer CAVI. These findings, along with the fact that men have a relatively lower total fat percentage, suggested us to ascribe more gender differences in cardiometabolic risk factors to the pattern of FM distribution rather than FM content. In both genders, %TFM was negatively associated with Matsuda index but %LFM was positively associated independent of age/BMI/WC. After adjusting for %TFM and/or %LFM, gender differences in insulin resistance indices became significant. These results reinforced our findings that FM distribution exerts influence on the difference in insulin sensitivity between women and men. Further analysis (Table 3) indicated that the relation between FM distribution and insulin resistance was stronger in women than in men; in particular, DXA-derived %LFM was a stronger correlate of insulin sensitivity in women than in men independent of age/BMI/WC. Such findings were consistent with the discovery of LFM playing an important role in protecting women against insulin resistance in previous studies [15]. More importantly, our study is the first to suggest that favorable effects of LFM on insulin resistance may be more pronounced in women than in men.

In contrast to %LFM, a higher %TFM was related to a detrimental cardiometabolic profile indicated by a lower insulin sensitivity, an atherogenic lipoprotein phenotype, a higher PAI-1 and TNF-*α*, and a lower adiponectin level in both genders. Moreover, positive relations among %TFM, PAI-1, and RLP-C have been continuously demonstrating significance after adjusting for covariates in men but not in women, whereas no significant associations among %LFM, PAI-1, and RLP-C were observed in both genders. Men had higher plasma PAI-1 level than women, but the significance of such differences disappeared after %TFM was taken into consideration. An increased plasma PAI-1 level is a well-established CVD risk factor [29], and our data suggested that %TFM is associated with a detrimental cardiometabolic profile through increased PAI-1 levels. Moreover, this connection might be stronger in men than in women. An intriguing finding of our study was that the gender difference in RLP-C was overridden by the adjustment for %TFM, but not for %LFM. Relations between RLP-C and FM distribution parameters were consistently notable both before and after adjusting for multivariables in men, but not in women. A number of studies have suggested that an increased RLP-C is a risk factor for endothelial dysfunction [30] and premature CVD independent of TG and LDL-C [31], as well as a mediator linking obesity with CVD [32]. Therefore, it is reasonable to speculate that RLP-C may play a role in mediating harmful effects of TFM on metabolism, in particular, in male individuals.

The reasons for TFM and LFM to have opposite relations with cardiometabolic risk factors remain obscure. Some studies assumed that adipokines, FFA, and other metabolites released from visceral fat directly drain into portal circulation, where they can exert adverse effects on the hepatic management of insulin and lipids [33]. According to the study in [15], a large depot of LFM is simply indicative of a propensity to store more adipose tissue in the subcutaneous compartment and less in the abdominal visceral cavity. However, several findings [34–39] suggested that this may be an oversimplification. Assessment of insulin resistance in central obese individuals has shown that a lower level of insulin resistance was observed in those individuals who have both central and subcutaneous obesity, suggesting that subcutaneous fat does possess advantageous effects on insulin resistance to counter the adverse effects of visceral fat [12]. This notion is also consistent with that about the effects of thiazolidinedione treatment, which improves insulin sensitivity despite that increasing total FM primarily increases the subcutaneous fat depot [37]. Reduction in central FM, rather than in peripheral FM, along with one-year lifestyle modification, is associated with an improvement in cardiorespiratory fitness in men [40]. Various adipokines secreted by adipose tissue have been implicated in the relationship between subcutaneous fat and insulin resistance. Of particular interest is the reverse association between adiponectin and insulin resistance, and this relationship is dependent on the degree of obesity as well as on other risk factors related to metabolic syndrome [38]. Our data demonstrated that %LFM is positively related to plasma adiponectin levels but %TFM is reversely related. These findings are consistent with those of a report [39] that visceral adipose tissue measured by CT is negatively related to adiponectin, while subcutaneous adipose tissue is positively correlated. Furthermore, we found that relations of %TFM and %LFM with ISI in women are attenuated by adjusting for adiponectin (data not shown). This observation, along with other extant observations ([11], for example), suggested that adiponectin may exert influence on the relationship between FM distribution and insulin sensitivity, thus performing a critical role in mediating protective effects of LFM on insulin resistance.

There are several potential limitations in this study. First, the study design was cross-sectional and the nature of this study was observational; therefore, identified correlations may not imply causality. Second, DXA was not able to distinguish visceral FM from subcutaneous FM in the trunk region or intramuscular FM from subcutaneous FM in the leg region. It was thus not possible in the present study to further identify the different effects of TFM and LFM on cardiometabolic risk factors due to subcutaneous and visceral FM. Finally, the cohort was relatively homogenous without clinically overt CVD. Consequently, the relationship between FM distribution and direct indices of atherosclerosis such as CAVI and IMT might be underestimated. Although confounders such as cigarette smoking, alcohol drinking, and drug administration were controlled, it was still unknown whether the results could be extended to healthy younger adults or less healthy population.

## 5. Conclusions

To investigate whether the gender-specific pattern of fat distribution is related to gender differences in cardiometabolic risk factors, we conducted an association study of body composition and various cardiometabolic parameters in a Japanese-based population without clinically overt metabolic and cardiovascular diseases. We discovered that men have more TFM whereas women have more LFM. In addition, we found that TFM has deleterious effects on glucose and lipid metabolism, adipokines, and inflammation markers in both genders; on the other hand, LFM has advantageous effects on these factors. More importantly, our investigation provided evidence that %LFM is preferentially related to ISI in women and %TFM is particularly related to PAI-1 and RLP-C in men, which explained in part why men have a more atherogenic cardiometabolic profile than women. Another finding of this study was that body fat distribution has no direct association with arterial stiffness and thickness, at least in relatively healthy middle-aged Japanese.

In summary, important findings in this study suggested that the gender-specific pattern of fat distribution contributes to gender differences in cardiometabolic risk factors. In our future study, we will continue this research direction and explore various mechanisms involved in the regulation of metabolic and cardiometabolic risk factors impacted by different fat distribution patterns.

## Figures and Tables

**Table 1:** Clinical and cardiometabolic characteristics of female and male participants.

	Women	Men	Model 1	Model 2	Model 3	Model 4
	(*n* = 148)	(*n* = 59)	*P*	*P*	*P*	*P*
Age (year)	49.8 ± 3.6	51.8 ± 4.8	<0.001	NS	NS	NS
Body weight (kg)	53.9 ± 7.0	69.2 ± 15.2	<0.001	<0.001	<0.001	<0.001
BMI (kg/m^2^)	22.0 ± 2.8	24.1 ± 5.3	<0.001	NS	NS	NS
Waist circumference (cm)	78.7 ± 8.1	85.9 ± 6.8	<0.001	NS	NS	NS
%total FM	30.1 ± 7.3	22.8 ± 6.2	<0.001	<0.001	<0.001	<0.001
%TFM	53.3 ± 5.0	62.7 ± 4.3	<0.001	—	—	—
%LFM	33.6 ± 5.0	26.5 ± 4.3	<0.001	—	—	—
%arm FM	9.0 ± 2.4	6.9 ± 1.5	<0.001	<0.001	<0.001	<0.01
HOMA-IR	1.21 ± 0.71	1.35 ± 0.91	NS	<0.05	NS	NS
Matsuda index	10.45 ± 5.05	11.49 ± 8.10	NS	<0.001	<0.001	<0.001
Triglyceride (mmol/L)	0.92 ± 0.40	1.65 ± 1.73	<0.001	NS	NS	NS
HDL cholesterol (mmol/L)	2.00 ± 0.41	1.51 ± 0.37	<0.001	<0.001	<0.001	<0.001
LDL cholesterol (mmol/L)	3.37 ± 0.77	3.17 ± 0.94	NS	NS	NS	NS
sdLDL (mg/dL)	17.48 ± 9.47	32.22 ± 18.15	<0.001	<0.01	<0.001	<0.01
ApoA1 (mg/dL)	178 ± 22	157 ± 22	<0.001	<0.01	<0.001	<0.01
ApoB (mg/dL)	93 ± 19	97 ± 22	NS	NS	NS	NS
RLP-C (mg/dL)	3.9 ± 1.8	8.0 ± 12.1	<0.001	NS	<0.05	NS
FFA (*μ*Eq/L)	600 ± 228	550 ± 231	NS	NS	NS	NS
Preheparin LPL mass (ng/mL)	80.2 ± 21.8	54.7 ± 17.3	<0.001	<0.001	<0.001	<0.001
Leptin (ng/mL)	7.6 ± 4.9	3.6 ± 2.3	<0.001	<0.001	<0.001	<0.001
Adiponectin (*μ*g/mL)	11.8 ± 4.9	6.8 ± 3.4	<0.001	<0.05	<0.01	<0.05
PAI-1 (ng/mL)	23.9 ± 15	40.1 ± 26.9	<0.001	NS	<0.01	NS
LogCRP	1.41 ± 0.53	1.02 ± 0.63	<0.001	<0.001	<0.001	<0.001
TNF-*α* (pg/mL)	0.77 ± 0.38	0.75 ± 0.35	NS	NS	NS	NS
8-epi-PGF_2*α*_ (pg/mg creatinine)	367.1 ± 185.8	368.3 ± 190.3	NS	NS	NS	NS
SBP (mmHg)	121 ± 16	134 ± 20	<0.001	NS	<0.05	NS
DBP (mmHg)	74 ± 11	85 ± 12	<0.001	<0.01	<0.001	<0.01
CAVI	7.00 ± 0.72	7.70 ± 1.07	<0.001	<0.01	<0.01	<0.01
Ave IMT (mm)	0.55 ± 0.11	0.59 ± 0.12	NS	NS	NS	NS
Max IMT (mm)	0.59 ± 0.11	0.64 ± 0.16	NS	NS	NS	NS

Data are mean ± SD. FM: fat mass; sdLDL: small, dense LDL; RLP-C: remnant-like particle cholesterol; LPL: lipoprotein lipase; 8-epi-PG_2*α*_: urinary 8-epi-PGF_2*α*_; SBP and DBP: systolic and diastolic blood pressure; CAVI: cardio-ankle vascular index; Ave IMT and Max IMT: average and maximum intima-media thickness of the common carotid artery; NS: no significance. Model 1: tested by the Mann–Whitney *U* test; model 2: adjusted for %TFM; model 3: adjusted for %LFM; model 4: adjusted for %TFM and %LFM.

**Table 2:** Gender-specific multiple regression analyses for %TFM and %LFM with cardiometabolic risk factors.

Dependent variables	Independent variables	Women				Men			
		Model 1		Model 2		Model 1		Model 2	
		*β*	SE	*β*	SE	*β*	SE	*β*	SE
HOMA-IR	%TFM	**0.365** ^‡^	0.077	**0.185** ^∗^	0.085	0.231	0.131	0.102	0.122
	%LFM	−**0.358**^‡^	0.077	−**0.177**^∗^	0.084	−0.246	0.130	−0.111	0.122
Matsuda index	%TFM	−**0.582**^‡^	0.105	−**0.528**^‡^	0.132	−**0.302**^∗^	0.128	−**0.237**^∗^	0.142
	%LFM	**0.617** ^‡^	0.095	**0.582** ^‡^	0.117	**0.288** ^∗^	0.129	**0.225** ^∗^	0.143
Triglyceride	%TFM	**0.291** ^‡^	0.081	**0.199** ^∗^	0.093	**0.427** ^†^	0.122	**0.406** ^†^	0.134
	%LFM	−**0.286**^‡^	0.081	−**0.187**^∗^	0.092	−**0.426**^†^	0.122	−**0.396**^†^	0.135
HDL-C	%TFM	−**0.268**^†^	0.081	−0.128	0.090	−0.248	0.129	−0.155	0.137
	%LFM	**0.253**†	0.082	0.110	0.089	**0.313** ^∗^	0.127	0.217	0.135
LDL-C	%TFM	0.157	0.083	0.089	0.094	−0.169	0.132	−0.187	0.150
	%LFM	−**0.219**^†^	0.082	−0.171	0.091	0.193	0.132	0.221	0.149
sdLDL	%TFM	**0.260** ^†^	0.094	0.074	0.107	0.217	0.191	0.322	0.220
	%LFM	−**0.217**^∗^	0.099	−0.018	0.108	−0.189	0.191	−**0.288**^∗^	0.214
ApoA1	%TFM	−**0.173**^∗^	0.083	−0.028	0.093	−0.040	0.134	0.027	0.149
	%LFM	**0.166** ^∗^	0.083	0.022	0.091	0.101	0.133	0.038	0.149
ApoB	%TFM	**0.285** ^†^	0.081	**0.188** ^∗^	0.092	0.150	0.132	0.087	0.143
	%LFM	−**0.336**^‡^	0.080	−0.251^†^	0.089	−0.142	0.132	−0.067	0.143
RLP-C	%TFM	0.082	0.084	0.107	0.098	**0.396** ^†^	0.124	**0.374** ^†^	0.137
	%LFM	−0.125	0.084	−0.160	0.096	−**0.385**^†^	0.124	−**0.352**^∗^	0.139
FFA	%TFM	−0.022	0.085	0.044	0.099	**0.324** ^∗^	0.127	0.204	0.140
	%LFM	−0.042	0.085	−0.126	0.096	−**0.297**^∗^	0.128	−0.176	0.141
Preheparin LPL mass	%TFM	−0.071	0.084	−0.059	0.099	−0.242	0.131	−0.136	0.126
	%LFM	0.045	0.084	0.024	0.097	**0.327** ^∗^	0.127	0.205	0.125
Leptin	%TFM	**0.333** ^‡^	0.080	−0.023	0.070	0.137	0.132	−0.064	0.116
	%LFM	−**0.318**^‡^	0.080	0.029	0.069	−0.202	0.131	0.005	0.117
Adiponectin	%TFM	−**0.394**^‡^	0.076	−**0.401**^†^	0.134	−**0.357**^†^	0.124	−**0.308**^∗^	0.132
	%LFM	**0.375** ^†^	0.106	**0.331** ^∗^	0.132	**0.356** ^†^	0.124	**0.295** ^∗^	0.133
PAI-1	%TFM	**0.349** ^†^	0.111	0.064	0.133	**0.308** ^∗^	0.128	**0.302** ^∗^	0.144
	%LFM	−**0.254**^∗^	0.117	0.063	0.129	−**0.261**^∗^	0.130	−0.242	0.147
LogCRP	%TFM	**0.284** ^∗^	0.112	−0.026	0.132	0.144	0.132	0.123	0.150
	%LFM	−**0.291**^∗^	0.115	0.008	0.128	−0.178	0.131	−0.170	0.149
TNF-*α*	%TFM	**0.311** ^∗^	0.133	0.244	0.173	0.225	0.131	**0.300** ^∗^	0.147
	%LFM	−**0.368**^†^	0.135	−0.318	0.166	−0.160	0.133	−**0.228**^∗^	0.150
8-epi-PGF_2*α*_	%TFM	−0.038	0.139	−0.158	0.164	0.038	0.133	0.128	0.126
	%LFM	0.054	0.142	0.201	0.159	−0.053	0.133	−0.135	0.126
SBP	%TFM	**0.337** ^∗^	0.129	0.046	0.153	0.233	0.129	0.128	0.134
	%LFM	−**0.318**^∗^	0.133	−0.052	0.088	−0.248	0.128	−0.141	0.134
DBP	%TFM	**0.285** ^∗^	0.121	0.104	0.150	0.188	0.129	0.122	0.131
	%LFM	−0.237	0.125	−0.032	0.146	−0.161	0.130	−0.093	0.132
CAVI	%TFM	−0.105	0.171	0.208	0.213	0.138	0.134	0.125	0.137
	%LFM	0.047	0.156	−0.212	0.173	−0.124	0.135	−0.114	0.137
Ave IMT	%TFM	0.168	0.191	0.283	0.254	0.237	0.174	0.066	0.173
	%LFM	−0.079	0.169	−0.047	0.206	−0.309	0.164	−0.145	0.163
Max IMT	%TFM	0.217	0.192	0.213	0.250	0.198	0.176	0.021	0.177
	%LFM	−0.150	0.169	−0.051	0.202	−0.267	0.166	−0.102	0.166

In model 1, there is only one independent variable that refers to either %TFM or %LFM. In model 2, independent variables include age/BMI/WC as well as %TFM or %LFM. SE stands for standard error of *β*. Three different superscripts denote various ranges of *P* value of *β*: ^∗^*P* < 0.05, ^†^*P* < 0.01, and ^‡^*P* < 0.001. Cells where *β* is of statistical significance are highlighted with the bold font. Abbreviations for variables were the same as in Table 1.

**Table 3:** Bivariate correlation of %TFM and %LFM with Matsuda index, triglyceride, and adiponectin, as well as comparison of coefficients between women and men.

		*r*_women_	*r*_men_	*Z*-statistic	*P* value for *r*_women_ versus *r*_men_
Simple bivariate correlation					
Matsuda index	%TFM	−0.579	−0.299	−2.241	0.025
	%LFM	0.644	0.286	2.992	0.003
Triglyceride	%TFM	0.289	0.425	−0.994	0.321
	%LFM	−0.284	−0.424	1.020	0.308
Adiponectin	%TFM	−0.397	−0.359	−0.282	0.778
	%LFM	0.365	0.358	0.051	0.959

*r*_women_ and *r*_men_ represent Pearson's correlation coefficients in women and men. All the bivariate correlation coefficients were statistically significant (*P* < 0.05).

## Abbreviations

%LFM: The percentage of lower-body fat mass (lower-body fat mass divided by total fat mass)
%TFM: The percentage of trunk fat mass (trunk fat mass divided by total fat mass)
8-epi-PGF_2*α*_: Creatinine-indexed 8-epi-prostaglandin F_2*α*_
ApoA1: Apolipoprotein A-1
ApoB: Apolipoprotein B-100
BMI: Body mass index
CAVI: Cardio-ankle vascular index
CRP: C-reactive protein
CT: Computed tomography
CV: Coefficient of variation
CVD: Cardiovascular diseases
DBP: Diastolic blood pressure
DXA: Dual-energy X-ray absorptiometry
ELISA: Enzyme-linked immunosorbent assay
FFA: Free fatty acid
FM: Fat mass
HDL-C: High-density lipoprotein cholesterol
HOMA-IR: Homeostasis model assessment insulin resistance
hs-CRP: Highly sensitive C-reactive protein
IMT: Intima-media thickness
ISI: Insulin sensitivity index (Matsuda index)
LDL-C: Low-density lipoprotein cholesterol
LFM: Lower-body fat mass
LPL: Lipoprotein lipase
MRI: Magnetic resonance imaging
OGTT: Oral glucose tolerance test
PAI-1: Plasminogen activator inhibitor-1
PWV: Pulse wave velocity
RIA: Radioimmunoassay
RLP-C: Remnant-like particle cholesterol
SBP: Systolic blood pressure
sdLDL: Small, dense LDL-C
TC: Total cholesterol
TFM: Trunk fat mass
TG: Triglycerides
TNF-*α:*: Tumor necrosis factor-*α*
VIF: Variation inflation factor
WC: Waist circumference.
